# Hidden in plain sight: a new frog species of the genus *Blommersia* from the oceanic island of Mayotte, Comoros archipelago

**DOI:** 10.3897/zookeys.994.57012

**Published:** 2020-10-17

**Authors:** David R. Vieites, Sandra Nieto-Román, Marcos Peso Fernández, Javier H. Santos-Santos

**Affiliations:** 1 Integrative Biogeography and Global Change Group, Museo Nacional de Ciencias Naturales, Consejo Superior de Investigaciones Científicas (MNCN-CSIC), 28006 Madrid, Spain Museo Nacional de Ciencias Naturales Madrid Spain; 2 Department of Animal Biology, University of Barcelona, Avenida Diagonal 645, 08028, Barcelona, Spain University of Barcelona Barcelona Spain; 3 Science and Business Ltd. Calle Perú, 6, 28290 Las Rozas de Madrid, Madrid, Spain Science and Business Ltd. Madrid Spain

**Keywords:** Amphibia, Anura, *Blommersia nataliae* sp. nov., Comoros archipelago, Mantellidae, Mayotte

## Abstract

The amphibian fauna of the western Indian ocean volcanic island of Mayotte is currently constituted by two species belonging to two genera of the anuran family Mantellidae: *Blommersia transmarina* and *Boophis nauticus*. These were recently described after intense fieldwork on the herpetofauna of the island. We here describe a third new species of frog from Mayotte, based on morphological and molecular data, that occurs in sympatry with the others and was utterly unnoticed until now. Genetic analyses of the16S rRNA gene, including all described and several undescribed species of the genus *Blommersia* from Madagascar and Mayotte, confirms that the new species is the sister species of *Blommersia transmarina*. Both species show apparent morphological differences as well as different life histories, ecology and genetics that confirm *Blommersia nataliae***sp. nov.** as a new species. We propose an IUCN Red List status of Critically Endangered for *B. nataliae***sp. nov.**

## Introduction

The Mantellidae is a hyperdiverse family of frogs mostly endemic to Madagascar (Glaw and Vences 2006), with few representatives known from the Comoran island of Mayotte, an oceanic island separated from Madagascar by water depths of more than 3000 m. So far, one species of the genus *Boophis* and one species of the genus *Blommersia* were known from Mayotte (Vences et al. 2003), which have recently been described as *Blommersia transmarina* and *Boophis nauticus* (Glaw et al. 2019). Those species represent one of the best examples of dispersal across oceanic barriers, as their ancestors colonized Mayotte by oceanic dispersal from Madagascar circa 8–6 Mya (Vences et al. 2003; Crottini et al. 2012).

The genus *Blommersia* comprises eleven described frog species and constitutes a monophyletic radiation with at least six undescribed, genetically divergent lineages that may warrant species status, suggesting that the genus is likely more diverse than currently known (Vieites et al. 2009). Species in this genus are of small size and mostly terrestrial, occurring mainly in swamps, rice fields, paddy fields and other water bodies, which could be degraded, in open lands, as well as in dry and rain forests across Madagascar. They usually call during the night from perches, showing distinct advertisement calls that are diagnostic of each species, and some species are morphologically very similar. Like some Malagasy microhylids of the genus *Stumpffia*, which are miniaturized frogs of SVL from 8–9 mm (*S. contumelia*) to 28 mm (*S. staffordi*) (Rakotoarison et al. 2017), *Blommersia* species tend to miniaturization with the smallest species having snout-vent lengths (SVL) around 14–16 mm (*B. kely*, *B. sarotra*, *B. domerguei*) to ca. 26 mm (*B. wittei*) (Glaw & Vences, 2007). This trend towards miniaturization makes them a remarkable group to study diversification patterns and speciation mechanisms related to body size evolution.

In this context, the known species present on Mayotte offer an exciting opportunity to study the morphological and life history evolution of species that have evolved in isolation without the presence of other congeners for the last 5 or 6 million years. *Blommersia transmarina* evolved into a bigger body size than any other *Blommersia* from Madagascar, likely having undergone a process of moderate gigantism compared to the rest of *Blommersia* species (Santos-Santos et al. 2020). During fieldwork on the Comoran island of Mayotte to collect specimens of this *Blommersia* species, we discovered a second undescribed species of much smaller body size that occurs in syntopy. This raises several questions related to the origin and evolution of these two species. First, are they sister species or the result of two independent colonizations from Madagascar? Second, if they are sister taxa, how did the morphological and ecological diversification processes take place in isolation with empty niches available and without other potential frog competitors? In this study, we integrate morphological and genetic data to formally describe and name this new taxon, presenting the first data on its morphology, life history and ecology.

## Materials and methods

### Voucher collection

Individuals of *Blommersia transmarina* were located by tracking their calls during regular searches to localize calling males, as well as by visiting different types of water bodies and forests in Mayotte. Although this species calls during the night, the newly discovered one is much more secretive in terms of calling as no calls were ever heard in breeding places during reproduction. Individuals of the new species were found first on the forest ground mixed with *B. transmarina*, and later we discovered their breeding places where reproduction could be observed.

Individuals were euthanized using chlorobutanol, fixed in 90% ethanol, and preserved in 70% ethanol. Tissue samples were taken in the field and preserved in 99% ethanol or RNAlater for molecular studies. Preserved specimens are deposited at the Museo Nacional de Ciencias Naturales (**MNCN**), Madrid, Spain. Code DRV corresponds to D. Vieites’ field numbers.

### Morphological measurements

Morphological measurements were taken by D. Vieites, using a Vernier caliper, to the nearest 0.1 mm (Table 1): SVL (snout-vent length), HW (head width at its widest point), HL (head length measured from the tip of the snout to the maxillary commissure), ED (horizontal eye diameter), END (eye-nostril distance, measured from the anterior corner of the eye to the middle of the nostril), NSD (nostril-snout tip distance), NND (nostril-nostril distance), TD (horizontal tympanum diameter), HAL (hand length, measured from the base of the hand to the tip of the finger III), FORL (forelimb length, measured from the axilla to the tip of the finger III with forelimb extended), HIL (hind limb length, measured from the center of the cloaca to the tip of toe III), FOL (foot length, measured from the base of the inner metatarsal tubercle to the tip of toe IV), FOTL (foot length including tarsus), TIBL (tibia length, measured as the distance from the outer surface of the flexed knee to the heel/tibiotarsal inflection), FGL (femoral gland length), FGW (femoral gland width), FGD (minimal femoral gland distance from each other), and RHL (relative hindlimb length: point reached by the tibiotarsal articulation when the hindlimb is appressed along the body). RHL is coded as follows: when the hindlimb is appressed along the body the tibiotarsal articulation reaches the (1) anterior eye corner, (2) eye center, (3) between the eye and nostril, (4) nostril, (5) snout tip, (6) between the nostril and snout tip, and (7) passes the snout tip. Measurements of the type series of *B. wittei* were taken from Vences et al. (2010) and Pabijan et al. (2011).

**Table 1.** Measurements of *Blommersia nataliae* sp. nov. and *B. transmarina* for comparison. See methods for abbreviations. The holotype of *B. nataliae* sp. nov. is shown in bold letters.

Species	Locality	Field number	MNCN catalog	Sex	SVL	HW	HL	TD	ED	END	NSD	NND	FORL	HAL	HIL	FOTL	FOL	TIBL	FGL	FGW	FGD	RHL
*B. nataliae* sp. nov.	Mont M’Sapere	DRV6854	MNCN50447	F	19.6	7.4	7.2	1.7	2.9	2	1.6	2.7	14	5.5	36	16	10	11				3
	Mont M’Sapere	DRV6855	MNCN50448	F	19.8	7.7	8.1	1.9	2.9	2	1.7	2.9	15	6	37	16	11	11				3
	Mont M’Sapere	DRV6869	MNCN50449	F	20.0	8.1	8.6	1.6	2.6	2	2	2.8	14	6	37	16	10	12				4
	Mont M’Sapere	DRV6868	MNCN50450	F	22.0	7	8.8	1.4	2.9	1.8	1.6	2.8	14	5.8	38	17	11	12				3
	Mont M’Sapere	DRV6808	MNCN50451	F	23.0	7.3	8.7	1.5	2.4	2.1	1.8	2.9	15	6.2	38	18	12	12				4
	Mont M’Sapere	DRV6862	MNCN50452	M	17.9	5.9	7	1.4	2.5	1.7	1.3	2.6	11	5	33	14	8.8	10	2	1.2	2.6	5
	Mont M’Sapere	DRV6857	MNCN50453	M	18.4	6.9	7.5	1.6	2.6	1.9	1.4	2.4	13	5.4	31	14	8.5	9.9	2.4	1.3		3
	Mont M’Sapere	DRV6861	MNCN50454	M	18.4	7.3	7.8	1.7	2.3	2	1.6	2.5	14	5.6	35	16	10	11	2.6	1.2		5
	Mont M’Sapere	DRV6859	MNCN50455	M	18.5	6.5	7.1	1.5	2.2	1.8	1.2	2.2	14	5.7	35	16	10	11	2	1.1		5
	**Mont M’Sapere**	**DRV6867**	**MNCN50456**	**M**	**18.6**	**6.3**	**7.5**	**1.5**	**2.8**	**2**	**1.4**	**2.4**	**14**	**5.4**	**33**	**15**	**9.7**	**10**	**3**	**1.8**	**2.3**	**3**
	Mont M’Sapere	DRV6863	MNCN50457	M	19.0	6.6	7.7	1.9	2.7	2.2	1.5	2.4	14	5.8	38	17	11	11	2.6	1.1		5
	Mont M’Sapere	DRV6860	MNCN50458	M	20.5	6.8	7.4	1.5	2.9	1.9	1.5	2.6	13	5.5	37	17	9.9	12	2.7	1.1	2.7	4
*B. transmarina*	Mont Choungi	DRV6835	MNCN50430	F	29.3	11	12	2.4	4	2.8	1.9	3.7	21	8.8	61	26	17	18				7
	Mont Combani	DRV6848	MNCN50431	F	26.0	9.1	10	2.4	3.5	2.7	1.9	3.3	20	7.8	52	22	14	16				7
	Mont Combani	DRV6805	MNCN50432	F	29.1	10	12	2.1	4.1	2.5	2	3.2	22	8.9	56	25	17	17				7
	Mont M’Sapere	DRV6813	MNCN50433	F	30.4	11	13	2.8	4.3	2.9	2	3.6	23	9.9	60	26	18	19				7
	Mont Bénara	DRV6831	MNCN50434	M	25.5	9.8	9	2.6	3.6	2.5	1.8	3	21	8.2	48	22	14	15	4.6	1.7		7
	Mont Bénara	DRV6832	MNCN50435	M	25.5	8.5	9.8	2.4	3.1	2.5	1.6	2.9	20	8.6	50.9	22	14	15	4.5	1.6	1.5	7
	Mont Bénara	DRV6833	MNCN50436	M	27.5	9.1	10	2.1	3.4	2.7	1.8	3	19	8.1	53	23	15	16	4.9	1.7		7
	Mont Bénara	DRV6841	MNCN50437	M	24.6	8.7	11	2.3	3.7	2.5	2.2	3.1	20	8.1	49	21	15	15	4.8	1.6	1	7
	Mont Bénara	DRV6838	MNCN50438	M	25.5	8.8	9.3	2.8	3.2	2.6	1.9	3	17	7.5	49	23	15	14	5	1.6		7
	Mont Bénara	DRV6836	MNCN50439	M	26.0	9.3	9.9	2.5	3.6	2.6	1.7	3	17	7.6	45	22	14	15	5.6	1.7	1.6	7
	Mont Combani	DRV6852	MNCN50440	M	24.5	8.4	9.7	2.1	3.4	2.2	1.4	3	16	6.4	47	21	13	15	3.8	1.5		7
	Mont Combani	DRV6819	MNCN50441	M	25.0	8.4	10	2.7	3.4	2.2	1.7	3.2	18	7.4	44	20	13	14	4.4	2		7
	Mont Combani	DRV6818	MNCN50442	M	26.0	8.8	9.4	2.4	3.5	2.8	1.7	3	19	7.8	49	24	14	15	4.6	1.7		7
	Mont Combani	DRV6806	MNCN50443	M	26.5	8.7	9.2	2.2	3.7	2.6	1.9	3.3	15	7.8	51	23	15	15	5.7	1.8	1	7
	Mont Combani	DRV6849	MNCN50444	M	27.0	9.4	12	2.4	3.9	2.5	2	3.6	18	8.6	46	23	16	15	4.5	1.6	1.6	7
	Mont Combani	DRV6850	MNCN50445	M	27.0	9.4	10	2.4	3.4	2.5	1.9	3.4	18	8.3	44	24	16	16	4.8	1.6		7
	Mont M’Sapere	DRV6807	MNCN50446	M	29.0	9.7	11	2.4	3.9	2.5	2.2	3.5	20	8.5	50	22	14	16	5.3	2	1.1	7

### Molecular analysis

DNA was extracted, and a fragment of 489bp of the mitochondrial 16S rRNA gene was amplified using primers 16S-AL and 16S-BH (see Vences et al. 2005). PCRs were performed in 25 μL reactions using ca. 50 ng genomic DNA, 10 pmol of each primer, 15 nmol of each dNTP, 50 nmol additional MgCl2, and the Taq PCR buffer (10 mM Tris-HCl, pH 8.3, 50 mM KCl, 1.1 mM MgCl2, and 0.01% gelatin) and 1 U of standard Taq DNA polymerase. PCR conditions follow Vieites et al. (2006): an initial denaturation step at 94 °C for 90 s; 35 cycles at 94 °C for 30 s, annealing temperature two degrees below the melting temperature of each primer for 45 s, extension at 72 °C for 60 s; final extension of 10 min at 72 °C. PCR products were purified using spin columns in a robot before cycle sequencing. A 10 μL sequencing reaction included 1–2 μL of template, 1 μL of sequencing buffer, 2 μL of 2 pmol primer, 1.8 μL of ABI sequence mix (BigDye Terminator version 3.1 Sequencing Standard, Applied Biosystems) and 3.2–4.2 μL of water. The sequence reaction was 33 cycles of 10 s at 96 °C, 10 s at 50 °C and 4 min at 60 °C. These were subsequently resolved on a 3100 ABI automated sequencer. Sequences were aligned in Geneious v. 11.1.5 (https://geneious.com) using the Clustal-Wallis algorithm, and the alignment was corrected by eye resulting in a 492 bp alignment.

For phylogenetic analysis, we assembled a dataset of sequences for all *Blommersia* species available in GenBank that cover all described and several undescribed candidate species (Vieites et al. 2009; Vences et al. 2010; Pabijan et al. 2011). Maximum likelihood phylogenetic analysis was performed using the program RaxML (Stamatakis 2014) with 1000 bootstrap replicates and the GTR model. *Mantella laevigata* and *Guibemantis liber* were used as outgroups. Newly determined sequences' GenBank accession numbers correspond to the series MW025124–MW025133.

## Results

### Molecular phylogenetics

**Figure 1.**
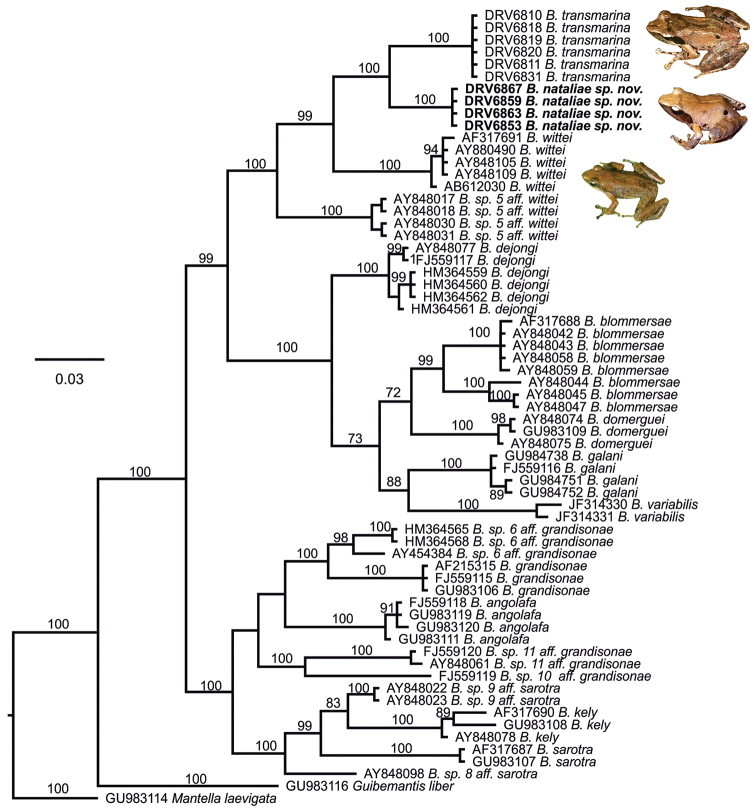
Maximum Likelihood phylogram of 16S rRNA relationships within *Blommersia*. Maximum Likelihood bootstrap support is shown above branches.

The recovered phylogenetic relationships between *Blommersia* species are shown in Fig. 1. We recovered three main clades within *Blommersia*: one constituted by *B. angolafa*, *B. grandisonae*, *B. sarotra* and *B. kely*, and five divergent undescribed lineages; the second constituted by *B. galani*, *B. dejongi*, *B. domerguei*, *B. blommersae* and *B. variabilis*; and a third clade constituted by *B. wittei*, an undescribed species resembling *B. wittei*, and two deep genetic lineages from Comoros. All the major clades had high bootstrap support with likelihood values higher than 99%. These two Comoroan lineages form a monophyletic group, having 4.3% divergence considering uncorrected pairwise 16S distances (21 substitutions), a value above the proposed 3% divergence in 16S typically used for recognition of distinct mantellid species (Vieites et al. 2009). One of them corresponds to the recently described *B. transmarina* as per sequence identity when compared to sequences provided by Vences et al. (2003) and Glaw et al. (2019). The other can be considered as a new candidate species, *Blommersia nataliae* sp. nov. This new candidate species shows 34 substitutions compared to *B. wittei* that results in an uncorrected pairwise divergence of 6.9 %, and there are 31 substitutions between *B. transmarina* and *B. wittei* that correspond to a 6.3% uncorrected pairwise distance.

### Taxonomy

**Figure 2.**
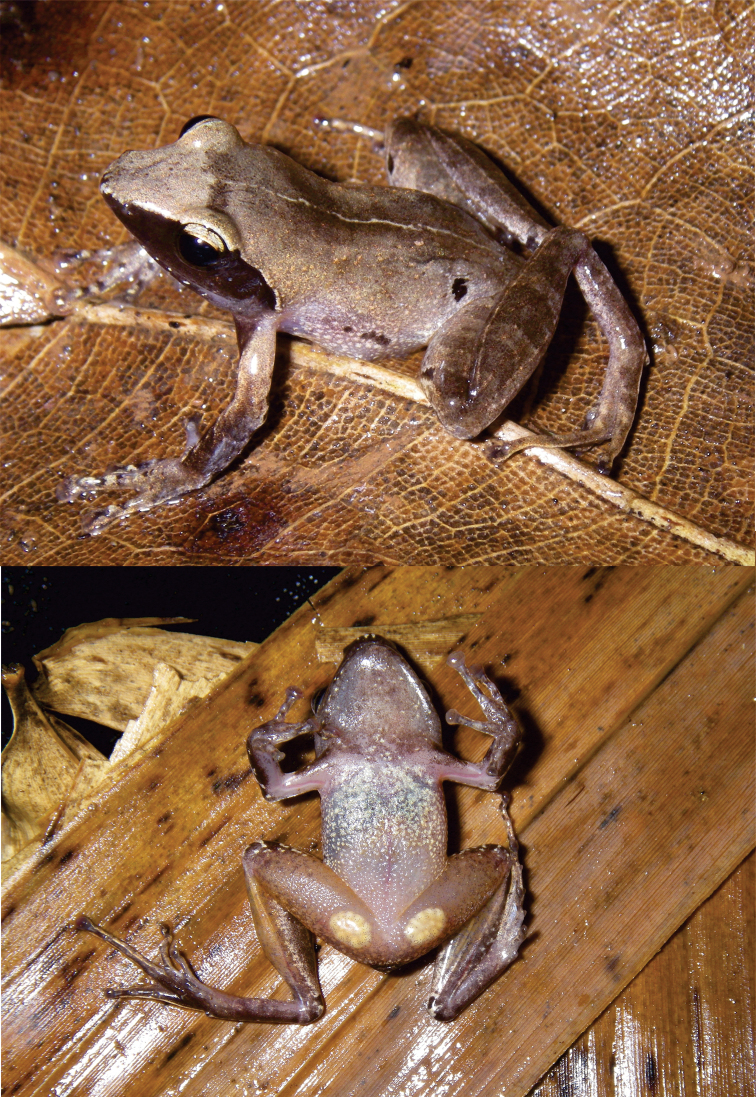
Dorsolateral and ventral views of the holotype of *Blommersia nataliae* sp. nov. DRV6867 (MNCN50456). Note the color and shape of the femoral glands which are a diagnostic character to distinguish it from its sister taxon *B. transmarina*.

**Figure 3.**
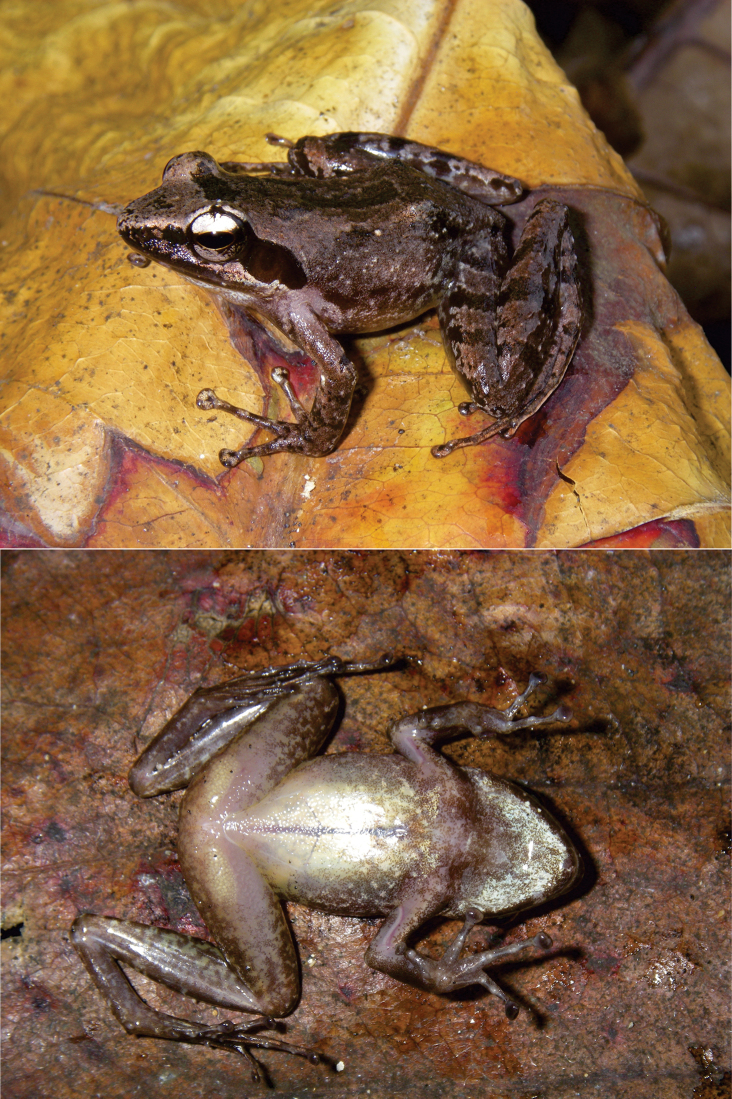
Dorsolateral and ventral views of *Blommersia transmarina* DRV6807 (MNCN50446), adult male collected at Mont M’Sapere in 2012 by D. Vieites and M. Peso Fernández. Note the shape and color of the femoral glands.

**Figure 4.**
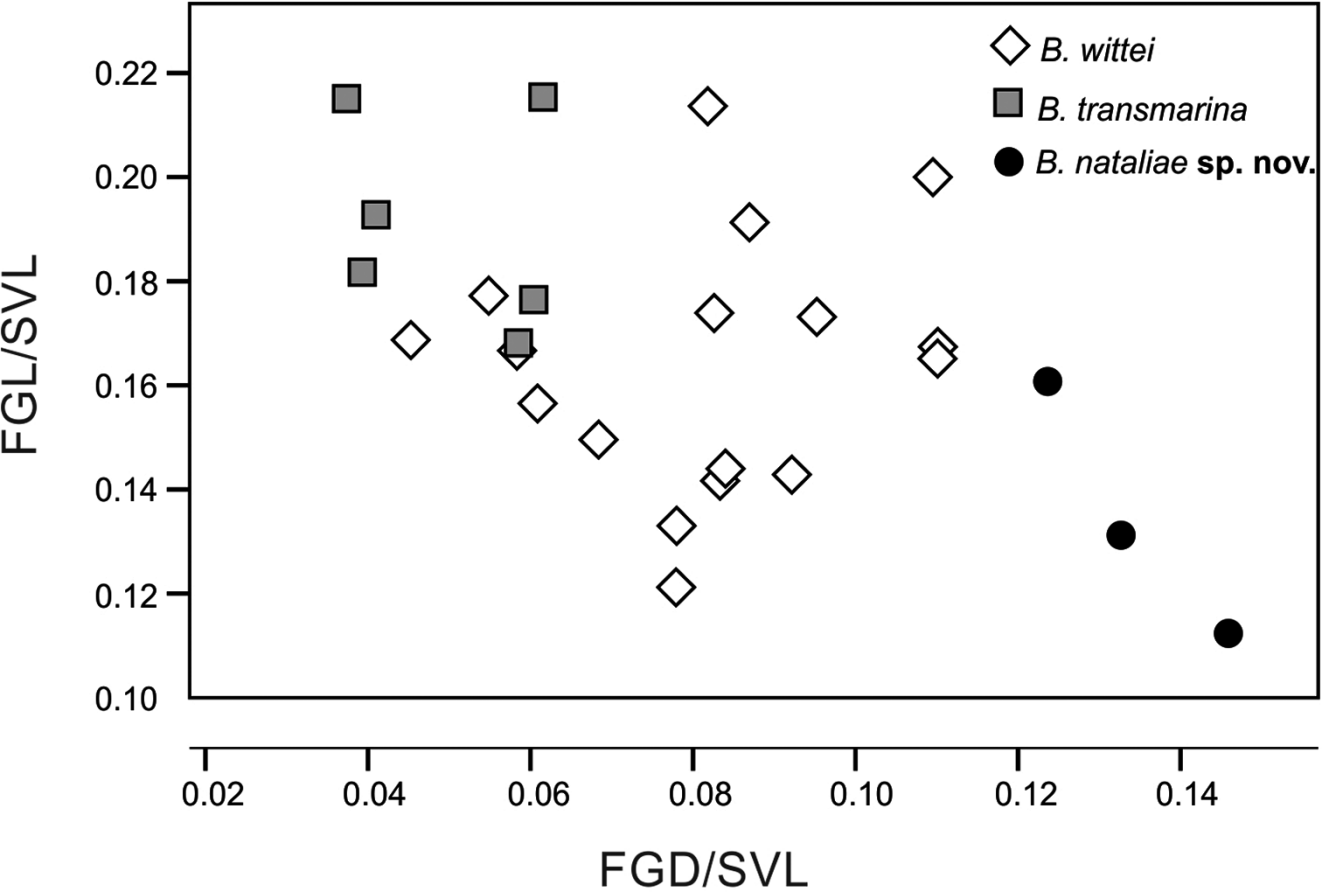
Scatterplot of relative femoral gland length (FGL/SVL ratio) and the relative distance between the inner edges of the femoral glands (FGD/SVL) in the two new species from Mayotte and their sister taxon *Blommersia wittei* from Madagascar. Measurements are based on Table 1; and for *B. wittei* on Vences et al. (2010) and Pabijan et al. (2011). Note the intermediate position of *B. wittei* between *B. transmarina* and *B. nataliae* sp. nov.

The two *Blommersia* lineages from Mayotte differ from each other, and from other *Blommersia* species, by several morphological traits: (1) the shape, position, and size of the femoral glands (Figs 2–4), (2) the presence or absence and shape of vomerine teeth, (3) tympanum size, (4) the extent to which lateral metatarsalia are fused or separated only by webbing and webbing formulae, (5) tongue shape, (6) relative hindlimb length, and (7) adult body size. The two *Blommersia* occur syntopically on Mayotte’s island and cannot be confused in the field with any other *Blommersia*.

Following an integrative taxonomic approach, morphological, genetic, life history, and biogeographic data support recognizing the newly discovered lineage on Mayotte as a new species under the evolutionary and biological species criteria. We therefore scientifically describe and name it here, providing a detailed description of adult morphology, intraspecific variation, and the first data on its life history and ecology.

#### Blommersia nataliae sp. nov.

Taxon classificationAnimaliaAnuraMantellidae

91B4828C-2611-5107-BE98-F2D0ED179A84

http://zoobank.org/8D15676C-402C-417E-B185-5551C356CA44

##### Holotype.

An adult male, left thigh muscle removed for genetic analyses. Original field number: “DRV6867, David R. Vieites collection”. Museo Nacional de Ciencias Naturales catalog number: MNCN50456. Collected in a degraded forest with giant bamboo, in forest leaf litter at the Mont M’Sapere, island of Mayotte (French Overseas Department), Comoros archipelago, -12.7656°S, 45.1852°E 500 m a.s.l. the 25^th^ November 2012 by D. Vieites and M. Peso Fernández.

##### Paratypes.

Females DRV6808 (MNCN50451), DRV6854 (MNCN50447), DRV6855 (MNCN50448), DRV6868 (MNCN50450), DRV6869 (MNCN50449); males DRV6857 (MNCN50453), DRV6859 (MNCN50455), DRV6860 (MNCN50458), DRV6861 (MNCN50454), DRV6862 (MNCN50452), DRV6863 (MNCN50457), collected at the type locality at the Mont M’Sapere in 2012 by D. Vieites and M. Peso Fernández.

##### Etymology.

Noun in the genitive case. D. Vieites and S. Nieto dedicate this species to their daughter Natalia Vieites Nieto, who has a birthmark resembling the beautiful conspicuous round moon-like brown spot characteristic of the species.

##### Diagnosis.

Assigned to the genus *Blommersia* in the family Mantellidae and subfamily Mantellinae by a combination of (1) presence of femoral glands and absence of nuptial pads in males, (2) presence of intercalary elements between ultimate and penultimate phalanges of fingers and toes (verified by external examination and microCT scanning), (3) presence of a single subgular vocal sac in males, (4) small size (adult SVL < 30 mm), and (5) molecular data.

Within the genus *Blommersia*, *B. nataliae* sp. nov. is characterized by the following unique suite of morphological characters: (1) small adult body size (SVL 18–23 mm), (2) round femoral glands that are distantly separated in males, (3) inconspicuous vomerine teeth, (4) ovoid tongue, (5) tibiotarsal articulation reaching between the eye and the nostril when adpressed along the body. Furthermore, the new species is differentiated from all other species of *Blommersia* by a significant molecular genetic differentiation (≥ 4.3% uncorrected pairwise-distance in 16S).

*Blommersia nataliae* sp. nov. can be distinguished from all other described *Blommersia* species except *B. wittei* and *B. transmarina* by the presence of vomerine teeth (vs. absence) and having separated metatarsalia (vs. unseparated).

*Blommersia nataliae* sp. nov. can be distinguished from its syntopic sister taxon *B. transmarina* by its rounded, distantly separated femoral glands (versus oblong, less separated glands; Figs 2–3). Fig. 4 represents the relative femoral gland length versus the relative distance between the femoral glands’ inner edges for both species and their sister taxon from Madagascar, *B. wittei*. *B. wittei* has an intermediate position between *B. nataliae* sp. nov. and *B. transmarina*; where *B. nataliae* sp. nov. presents rounded and shorter glands that are ca. two times more separated between each other than in *B. transmarina* (median FGD 2.6±0.2 mm vs. 1.3±0.3 mm, respectively) and ca. half shorter (median FGL 2.6±0.4 mm vs. 4.8±0.5 mm) (see also Figs 2–3). *Blommersia nataliae* sp. nov. also differs from *B. transmarina* in having inconspicuous vomerine teeth versus well developed and showing a V shape, ovoid tongue (vs. bifid), shorter hindlimbs with tibiotarsal articulation reaching between the eye and the nostril (vs. surpassing well the snout when appressed along the body), less distinct inner metatarsal tubercle, webbing formula [1(1), 2i(1.75), 2e(1), 3i(2.5), 3e(2), 4i/e(3), 5(1.5) versus 1(1), 2i(1–1.5), 2e(0.5), 3i(1.5), 3e(1), 4i(2–2.5), 4e(1.5–2), 5(0.5)], and by showing a brown facial mask from the snout, under the loreal region, to the tympanum, and by the presence of (usually) one very conspicuous moon-like spot on the back of each flank, close to the pelvic region and the hindlimbs. From *B. wittei*, it differs in femoral gland dimensions and position (see Fig. 4), ovoid tongue (vs. bifid), slightly longer hindlimbs with tibiotarsal articulation reaching between the eye and the nostril (vs. reaching the anterior corner of the eye), less distinct inner metatarsal tubercle, webbing formula, and in coloration. *Blommersia wittei* has a proportionally smaller tympanum than both *B. nataliae* sp. nov. and *B. transmarina* (mean ratio TD/SVL 0.068±0.007, vs. 0.081±0.009 in *B. transmarina* and 0.080±0.010 in *B. nataliae* sp. nov.).

**Description of the holotype (Fig. 2).** Male specimen in good state of preservation. Part of the left thigh taken for genetic analyses. SVL = 18.6 mm. The body is slender; the head is slightly longer than wide but not wider than the body. Snout slightly pointed and rounded in lateral views with protuberant nostrils directed laterally, nearer to the tip of snout than to eye; canthus rostralis indistinct and straight; loreal region straight; tympanum distinct and rounded, with a diameter of 60% of the eye diameter; supratympanic fold present and slightly distinct behind the tympanum, but indistinct in its anterior part between the eye and the tympanum; tongue slender and ovoid, slightly notched posteriorly but not bifid; vomerine teeth present but very inconspicuous and very small, hard to see, and not grouped; maxillary teeth rudimentary; choanae rounded. The arms are slender with distinct, single subarticular tubercles, the inner and outer metacarpal tubercles distinct, the fingers without webbing, and the relative length of the fingers is 1<2<4<3; terminal finger discs are enlarged and nuptial pads absent. Hindlimbs are relatively robust; the tibiotarsal articulation reaches between the eye and the nostril when the hindlimb is appressed along the body; the lateral metatarsalia are separated; the inner metatarsal tubercle is small and the outer distinct; toe discs are enlarged, and the webbing between toes weakly developed [1(1), 2i(1.75), 2e(1), 3i(2.5), 3e(2), 4i/e(3), 5(1.5)]. The skin on the dorsal surface is smooth without folds or ridges. The ventral skin is uniformly smooth. Femoral glands are very distinct in life, as well as after ethanol preservation, in external view.

Coloration of the Holotype (Fig. 2). In life, the overall color is creamy light brown with golden spots on the flanks, arms, and legs. It shows a thin yellowish line from the midpoint between the eyes to the vent. The legs are slightly darker brown and bands are visible. It shows a dark brown spot on the flanks and a characteristic larger moon-like spot on each flank’s back close to the pelvic region and the hindlimbs. It presents a dark brown facial mask that covers from the snout, under the loreal region, to the tympanum (see Fig. 2). The loreal region, as well as the outer iris periphery, shows a thin golden-colored line. The pupil is black and the inner iris area dark brown, while the outer iris area is golden with dark reticulations. The throat is brownish. The belly is light brown with some whitish, silver, and gold spots. The femoral glands are oval with a yellowish coloration and 9–10 circular internal rounded structures. After eight years in preservative, the back shows a creamy brown coloration that gets lighter towards the sides of the body, but the golden spots and dorsal line are lost. Ventral coloration is light brown without evident golden spots. The moon-like brown spot in the posterior part of the flanks is still evident, as well as the small ones on the flanks behind the arms. The femoral glands are whitish.

##### Variation.

The measurements of the holotype and paratypes are provided in Table 1. Sexual dimorphism is apparent in several characters: males present distinctive femoral glands, females are larger than males [males: median±SD SVL= 18.5±0.8 mm (min-max=17.9–20.5); females: 20±1.5 mm (min–max=19.6–23 mm)]. The color pattern is rather homogeneous, but females show an overall much creamier coloration than males, which are slightly darker. Both males and females show the characteristic brown rounded moon-like spot on the posterior flanks of the body, as well as some blotches on the lateral body sides behind the arms of variable size and shape. Vomerine teeth are more evident in specimen DRV6854 (MNCN50447), but only on one side of the vomer, and in DRV6855 (MNCN50448) and DRV6808 (MNCN50451) on both sides and more evident than in the holotype. Female DRV6855 (MNCN50448) lacks the moon-like blotch in the posterior side of the body, but shows a large circular one behind the arms. Female DRV6808 (MNCN50451) shows a similar pattern, but with a smaller blotch. Female DRV6868 (MNCN50450) shows a constellation of small rounded to irregular dark blotches from behind the arms to the inguinal region.

##### Natural history of *Blommersia nataliae* sp. nov.

**Figure 5.**
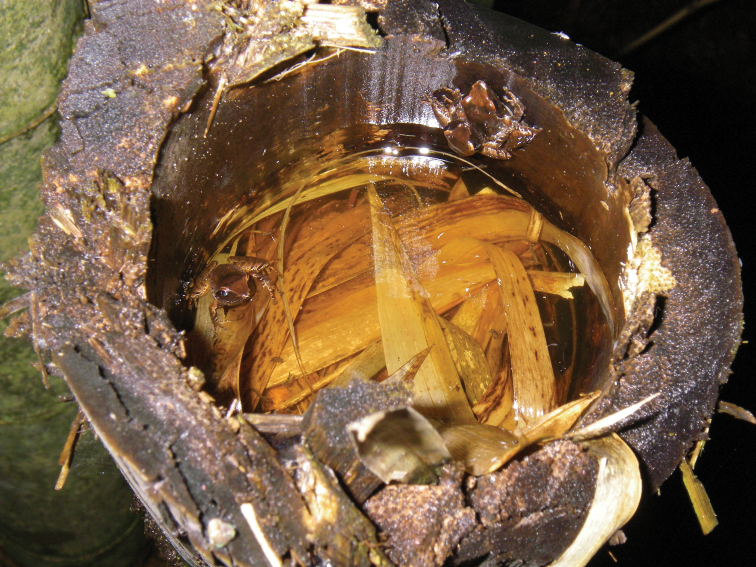
Photo of three specimens of *Blommersia nataliae* sp. nov. during reproduction in a cut bamboo trunk filled with water. On top of the picture, a male can be seen on top of a female, and another male is in the water on the left side of the trunk.

The species was found on the ground and in its breeding places: cut bamboo trunks filled with water (Fig. 5). There, we observed several males waiting for females to reproduce the night of 28^th^ November 2012, with a temperature of 24.6 °C. No frogs were seen in the breeding places during the day. No call was ever heard during reproductive periods despite several attempts and leaving a digital recorder running for two hours at a breeding spot with active frogs at night, while *B. transmarina* and *B. nauticus* were calling. The clutches were placed on the bamboo’s inner walls above the water, but only a few eggs seemed to be fertilized and showed embryonic development. We counted three clutches of 42, 43, and 22 eggs on the walls of cut bamboo trunks in November 2012 at Mont M’Sapere. Females seem to deposit several unfertilized eggs in the water that can serve as food for the tadpoles, but more research is needed to disentangle the species’ reproductive strategy. Individuals were seen during the day on the ground in the forest leaf litter, mixed with *B. transmarina*. No frogs were ever seen in other microhabitats like swamps, ponds, streams, or similar water bodies, where *B. transmarina* reproduces. The species reproduces during the rainy season if the bamboo holes are filled with water. We observed clutches and tadpoles in November–December 2012 and April 2014. In some years with little rain (e.g., November 2019), we observed the frogs, but all usual reproduction sites were empty of water with no clutches or tadpoles. The scarcity of rain may strongly affect this species in the near future, limiting its possibility to reproduce.

##### Distribution.

Initially found on the slopes of Mont M’Sapere only where there is still forest present and giant bamboos, between 235 m a.s.l. and 409 m a.s.l. (2012, 2014, and 2019). In the 2014 expedition, we also found the new species at Mont Bénara (12.8712°S, 45.15614°E, 317 m a.s.l.) in a forested place with fewer bamboo stands available, but few specimens. It is possible that the species breeds in other microhabitats (e.g., tree holes) as in the places where it occurs at Mont Bénara there are not many bamboo stands available, but this hypothesis needs to be confirmed. After several trips and visits around the whole entire main island of Mayotte and surrounding islets, with very intense fieldwork, we have not found it anywhere else, and the habitat appears to be degraded for the species.

##### Conservation.

The new species is only known from two localities and seems restricted to mountain areas where the forest is still present with giant bamboo stands. The area of occupancy is estimated to be less than 10 km^2^ from the elevation range where it was observed (235 to 409 m a.sl.) and the remaining forest available at the Reserve Forestière de Majimbini (Mont M’Sapere) and the Reserve Forèstiere du Mont Benara (Dupuy et al. 2019). There are bamboo stands also at lower elevations in degraded forests, but we never detected the species there. The distribution range is extremely small, the habitat is increasingly degraded, the breeding places (broken bamboo trunks) not frequent, and the fact that the observed densities seem to be very low suggest to consider the species as Critically Endangered according to IUCN criteria, in need of urgent conservation actions considering the ongoing degradation of these forest habitats. The impacts of local harvesting of bamboos or fires are not known, but it seems critical for the conservation of the species. The introduction of the chytrid fungus in Mayotte could decimate this species as well as *B. transmarina* in a few years. The consequences of climate change, such as a reduction in rainfall that fills its reproductive sites, may strongly affect the species as well.

## Discussion

For a long time, the Mantellidae was considered an endemic family of frogs to Madagascar, where it is highly diverse, with more than 220 species described so far. The presence of two amphibian mantellid species in Mayotte, a *Boophis* and a former *Mantidactylus* (now *Blommersia*), was known for a relatively long time (Vences et al. 2003; Glaw et al. 2019). A 2003 work showed that these represent different genetic lineages from Malagasy taxa (Vences et al. 2003), supporting the natural presence of mantellids in Mayotte, and suggesting two independent oceanic colonizations of Mayotte by oceanic dispersal. Despite intensive fieldwork in the Comoros archipelago (Hawlitschek et al. 2011), that materialized in a recently published distribution atlas of the amphibians and reptiles of Comoros (Augros 2019), nobody was aware until now of the presence of a third species of amphibian on Mayotte, which raises interesting questions about their origin and evolution in isolation from the rest of congeners in Madagascar.

Oceanic islands offer unique opportunities to study the evolution of ecological and morphological characters as new colonizers have few competitors and empty niches to exploit. In many cases, this leads to island radiations that show a wide range of ecological and morphological adaptations in small territories (e.g., Freed et al. 1987). There are not many cases of amphibian colonization of oceanic islands, being remarkable the case of the Guinean Gulf Islands where anurans and a caecilian were able to colonize from Africa (Measey et al. 2007; Bell et al. 2015a; 2015b), and the case of western Indian Ocean islands (Vences et al. 2003). The existence of two species of *Blommersia* frogs in Mayotte that are sister taxa and evolved in complete isolation from other *Blommersia* species offers a unique model system and opportunity to study the process of speciation from different perspectives, including the genetic, morphological, and ecological ones. Locals report small brown frogs in Mohéli, in forests north of Nioumachoua (G. Decalf pers. com.). Mohéli is another island of the Comoroan archipelago located west of Mayotte. This would suppose yet another oceanic dispersal event; however, these frogs have not been reported by locals for many years, and they are presumably extinct, pending further confirmation of its species identity and existence.

From the external morphological point of view, and comparing with their sister taxon *Blommersia wittei* from Madagascar (Table 1), *B. nataliae* sp. nov. and *B. transmarina* diverged in opposite directions. *Blommersia wittei* shows intermediate measurements between the two in most characters, being a typical small *Blommersia* species. *B. transmarina* has undergone a process of increase in body size, which can be considered towards a moderate gigantism compared to other *Blommersia*, being the largest *Blommersia* species known so far (Glaw et al. 2019; Santos-Santos et al. 2020). The overall aspect of *B. transmarina* more closely resembles other Mantellinae genera from Madagascar than a typical *Blommersia*, being bigger, more robust, and darker in coloration with proportionally longer and more robust legs. *Blommersia nataliae* sp. nov., on the contrary, has undergone a process towards miniaturization from their shared common ancestor, similar to other *Blommersia* species from Madagascar. We hypothesize that the ancestor of both species arrived in Mayotte and populations started to exploit available ecological niches, as they do not compete with the other frog species, *Boophis nauticus*, present on the island. In the ecological component, divergence happened in the selection of breeding habitats, with *B. transmarina* retaining the same ecology as *B. wittei*, but *B. nataliae* sp. nov. specializing in reproducing in bamboo trunks filed with water and maybe other cavities, leading to a specialized tadpole. Adaptation to different environments is also known from *Blommersia angolafa*, which is phytotelmic and reproduces in the water accumulated in fallen leaves (Andreone et al. 2010).

The different events of ecological and morphological release in Comoran species lead to their morphological divergence with an adult morphological pattern in *B. nataliae* sp. nov. that resembles other *Blommersia* from Madagascar that evolved towards miniaturization, whereas *B. transmarina* occupies the morphological space of other larger genera present in Madagascar (e.g., *Mantidactylus* or *Gephyromantis*). This offers a unique opportunity to explore the mechanisms of their morphological evolution in isolation.

In this study, we have followed an integrative taxonomic approach by incorporating morphological, genetic, and life history data, which supports the species status of this new taxon. Despite the effort to register mating calls from the new species, we never heard it in the field or in captivity, even when observing reproduction in front of us. This contrasts with *B. transmarina*, in which males are very active, calling at night everywhere, even at close distance. Hence, we could not include calls in the analyses, and the call of this species still needs to be determined.

The observed genetic divergence between Mayotte’s two *Blommersia* species is above the values observed between other mantellid sister species (Vences et al. 2005; Vieites et al. 2009), and the morphological differences are clear. Both species have evolved in isolation and occur on a small island, with the actual range of *B. nataliae* sp. nov. being minimal. This, united to its particular breeding ecology and requirements for reproduction, the lack of suitable areas for reproduction, and its apparent low density and small population size, suggest that this species is threatened. We propose a category of Critically Endangered following IUCN criteria and predict that the species can disappear in parallel to the degradation of its environment if its remaining pockets of habitat are not well managed. In the last eight years, we have observed a loss of part of the bamboos in mid-elevation forests that are the only known breeding habitat of this species. Not all cut bamboo trunks are suitable for the species as they cannot hold water, so the current available breeding habitat is very scarce. Although giant bamboo stands occur at lower elevations, *B. nataliae* sp. nov. is not present there, so the management of the forest seems critical to ensure the survival of the species. *Blommersia transmarina* shows a different conservation situation since it can profit from degraded habitats, but it is more common in mountains and forested habitats.

Two other significant threats can also affect the conservation of the Comoran herpetofauna apart from habitat degradation. Warming of the western Indian Ocean is becoming more intense, with a higher likelihood of extreme climatic events (Ng et al. 2015), like droughts or cyclons, in this century. In 2016 and 2017, a severe drought affected the Comoros archipelago and Madagascar, with limited amphibian reproduction during the rainy season as wetlands were dry and the rain was scarce (pers. obs.). This scarcity of rain can severely affect *B. nataliae* sp. nov. as the microhabitats where it reproduces need to be filled up with water to allow reproduction. However, it could be counteracted with inexpensive conservation actions like filling them with water manually.

In contrast, *B. transmarina* is present in the typical *Blommersia* habitats: swamps, ponds, little brooks, roadside channels, and even water deposits, fountains, or buckets, occurring in both forest habitats as well as in very degraded areas. The breeding season can extend throughout the year as long as there is rain, as we observed clutches in spring, fall, and winter, although the main reproductive activity coincides with the rainy season. In captivity, they can reproduce all year round. The males of *B. transmarina* are very active in breeding grounds, calling from leaves, shrubs, low branches of trees or rocks, sometimes with frenetic activity. They even were even found calling in fountains of hotels during the night, so the potential impact of climate change may be less intense for this species. The introduction of the chytrid fungus, which so far has not been reported in Mayotte, could easily drive the three amphibian species known on the island to extinction as well.

## Supplementary Material

XML Treatment for Blommersia nataliae

## Supplementary materials

Supplementary material 1 Data descriptor for *Blommmersia nataliae* sp. nov. Data type COL https://binary.pensoft.net/file/476546This dataset is made available under the Open Database License (http://opendatacommons.org/licenses/odbl/1.0/). The Open Database License (ODbL) is a license agreement intended to allow users to freely share, modify, and use this Dataset while maintaining this same freedom for others, provided that the original source and author(s) are credited.David R. Vieites, Sandra Nieto Román, Marcos Peso Fernández, Javier H. Santos-Santos
